# *In silico* and *in vitro* identification of inhibitory activities of sorafenib on histone deacetylases in hepatocellular carcinoma cells

**DOI:** 10.18632/oncotarget.21030

**Published:** 2017-09-16

**Authors:** Tsang-Pai Liu, Yi-Han Hong, Pei-Ming Yang

**Affiliations:** ^1^ PhD Program for Cancer Biology and Drug Discovery, College of Medical Science and Technology, Taipei Medical University and Academia Sinica, Taipei, Taiwan; ^2^ Department of Surgery, Mackay Memorial Hospital, Taipei, Taiwan; ^3^ Mackay Junior College of Medicine, Nursing and Management, New Taipei City, Taiwan; ^4^ Department of Medicine, Mackay Medical College, New Taipei City, Taiwan; ^5^ Liver Medical Center, Mackay Memorial Hospital, Taipei, Taiwan; ^6^ Graduate Institute of Cancer Biology and Drug Discovery, College of Medical Science and Technology, Taipei Medical University, Taipei, Taiwan

**Keywords:** connectivity map (CMap), hepatocellular carcinoma (HCC), histone deacetylase (HDAC), polypharmacology, sorafenib

## Abstract

Although sorafenib has been approved for treating hepatocellular carcinoma (HCC), clinical results are not satisfactory. Polypharmacology (one drug with multiple molecular targets) is viewed as an attractive strategy for identifying novel mechanisms of a drug and then rationally designing more-effective next-generation therapeutic agents. In this study, a polypharmacological study of sorafenib was performed by mining the next-generation Connectivity Map (CMap) database, CLUE (https://clue.io/). We found that sorafenib may act as a histone deacetylase (HDAC) inhibitor based on similar gene expression profiles. *In vitro* experimental analyses demonstrated that sorafenib indirectly inhibited HDAC activity in both sorafenib-sensitive and -resistant HCC cells. A cancer genomics analysis using the cBioPortal online tool showed the frequent upregulation of HDAC mRNAs. Furthermore, HCC patients with higher expressions of HDAC1 and HDAC2 had worse overall survival. Taken together, our study suggests that inhibition of HDAC by sorafenib may provide clinical benefits against HCC, and enhancement of HDAC-inhibitory activity of sorafenib may improve its therapeutic efficacy. In addition, our study also provides a novel strategy to study polypharmacology.

## INTRODUCTION

Hepatocellular carcinoma (HCC) is one of the most common cancers and a leading cause of cancer-related deaths worldwide [1]. Major remedial treatments for HCC are surgical resection and liver transplantation. However, only 15%∼25% of patients are suitable for these treatments [2]. In addition, HCC tends to develop chemoresistance and is highly refractory to chemotherapy. Furthermore, no effective therapy can be used on patients with advanced or metastatic disease [2]. Molecular targeted therapy is considered a new treatment option. Sorafenib, a multi-kinase inhibitor, was approved to treat advanced HCC in 2007 [3]. Unfortunately, it seems that sorafenib treatment does not achieve satisfactory results in HCC patients, because less than 3 months of actual survival time was gained in both Western and Asian populations [3, 4]. Thus, a better understanding of the action mechanism of sorafenib is urgently needed to improve its therapeutic efficacy.

Polypharmacology is one drug exhibiting actions on more than one molecular target [5]. Predicting the polypharmacology of clinical drugs provides an opportunity to improve their therapeutic efficacies through discovering novel action mechanisms [6]. In recent years, large-scale databases have continually been established to correlate drug-induced changes in gene or protein expressions with their phenotypes on a global scale [7]. Utilization of these databases will be highly useful in investigating polypharmacology. For example, the Connectivity Map (CMap) is a database containing gene expression profiles from cultured human cancer cells treated with small bioactive molecules. By mining and comparing gene-expression signatures, this tool can be used to find connections among small molecules sharing similar action mechanisms [8]. The Library of Integrated Cellular Signatures (LINCS), the next generation of CMap, generates gene expression signatures using the L1000 platform [9]. The L1000 assay is a messenger (m)RNA expression profiling technique based on a reduced representation of the genome whereby 1000 carefully selected transcripts are monitored, and from which the remainder of the transcriptome can be computationally inferred [9]. Compared to CMap, LINCS contains gene expression profiles of small molecules and also those of genetic constructs for knocking-down genes (short hairpin (sh)RNA) or over-expressing genes (complementary (c)DNA). LINCS has greater numbers of gene expression signatures (8870 perturbagens) and cell lines (nine cancer cell lines) than CMap, and thus may provide more-reliable predictions. Previously, LINCS was accessed through a web-based interface, lincscloud (http://www.lincscloud.org/), that was recently replaced by CLUE (https://clue.io/).

In this study, we mined the CLUE database to discover novel actions of sorafenib. We found that inhibition of histone deacetylase (HDAC) activity is a novel function of sorafenib. *In vitro* experimental analyses validated that sorafenib indirectly inhibits HDAC activity in both sorafenib-sensitive and -resistant HCC cells. A cancer genomics analysis indicated that higher mRNA expressions of HDAC1 and HDAC2 in HCC were associated with a worse overall survival of HCC patients. Therefore, inhibition of HDAC activity is associated with the anticancer activity of sorafenib against HCC.

## RESULTS

### CMap analysis predicts sorafenib as a potential HDAC inhibitor

**Figure 1:**
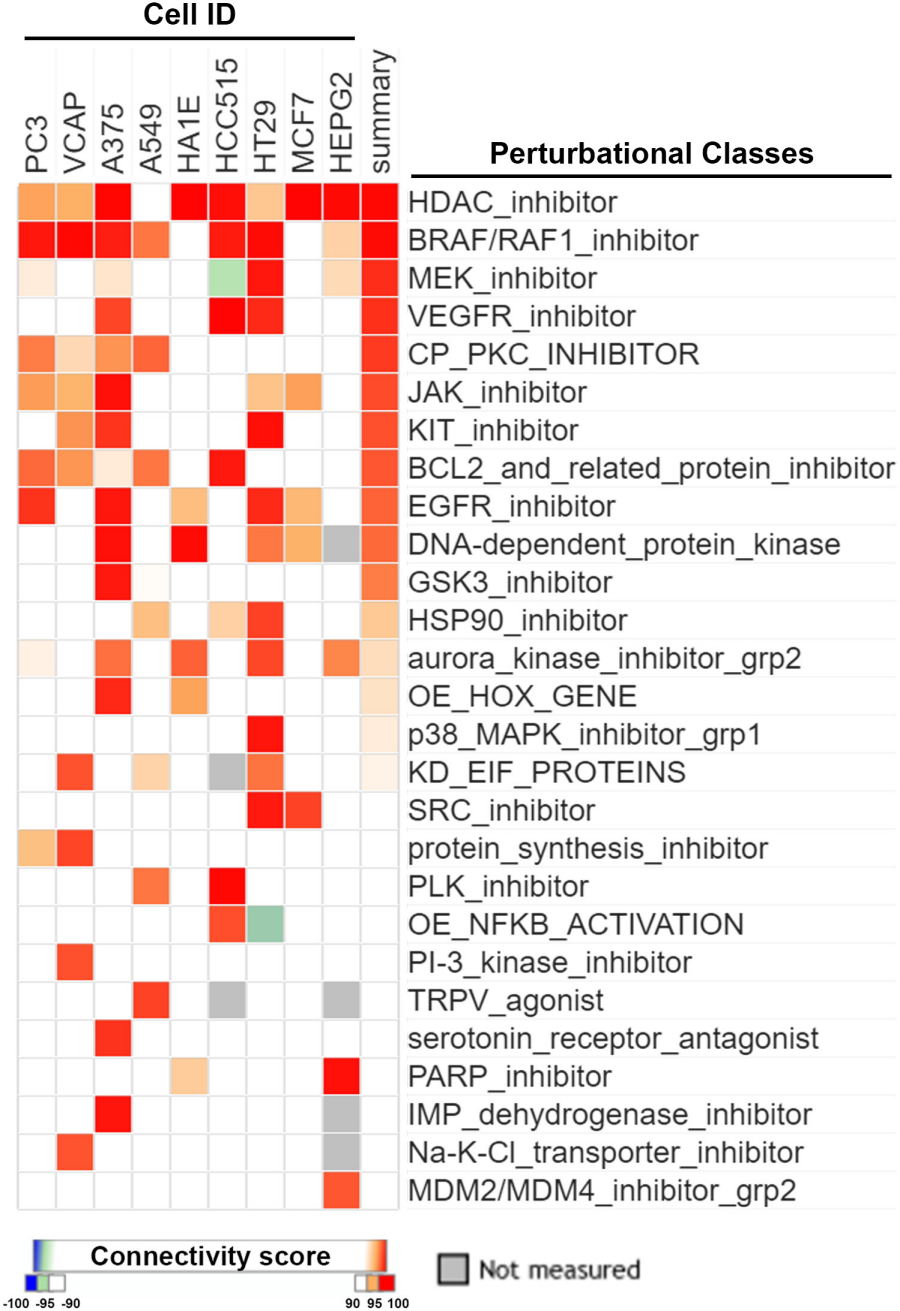
CMap analysis of drug connections with sorafenib Connections of sorafenib with other compounds were analyzed using an online Touchstone tool of the CLUE database as described in “Materials and Methods”. Drug connections were viewed as a heatmap. Perturbational classes were ranked based on summary connectivity scores.

**Figure 2:**
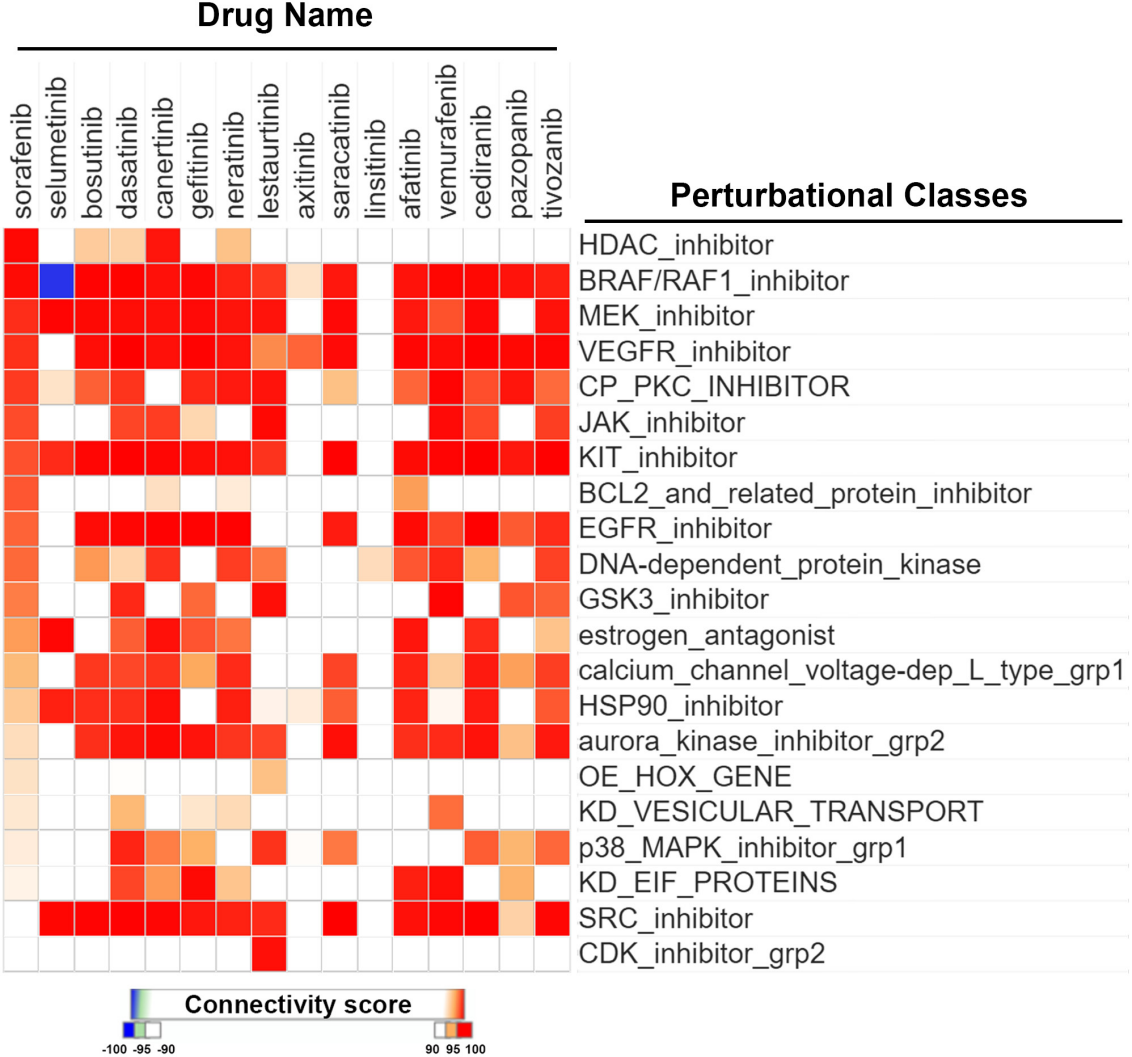
CMap analysis comparing drug connections with sorafenib and other protein kinase inhibitors Connections of sorafenib with other protein kinase inhibitors were analyzed using an online Repurposing tool of the CLUE database as described in “Materials and Methods”. Drug connections were viewed as a heatmap. Perturbational classes were ranked based on summary connectivity scores of sorafenib. Only the most similar perturbational classes and a summary of each drug are shown. The original figure is shown in Supplementary Figure 1.

It is believed that CMap is a suitable tool for discovering novel mechanisms of drugs by comparing similarities of gene expression profiles among drugs. By querying CLUE (https://clue.io/), the next generation CMap database, for the polypharmacology of sorafenib, we accidentally found similarities of HDAC inhibitors with sorafenib (Figure 1). Surprisingly, the gene expression profile of sorafenib was more similar to those of HDAC inhibitors compared to BRAF/RAF1, MEK, and vascular endothelial growth factor receptor (VEGFR) inhibitors (Figure 1). To investigate whether this is a general phenomenon of protein kinase inhibitors, we queried the CLUE database and compared drug similarities among sorafenib and other common protein kinase inhibitors (their names and functional descriptions are listed in Supplementary Table 1). As shown in Figure 2, the gene expression profile of canertinib (connectivity score = 99.3914), like sorafenib (connectivity score = 99.7771), was very similar to those of HDAC inhibitors. Other inhibitors, including bosutinib, dasatinib, and neratinib, also had higher connectivity scores (> 90). However, other compounds were not as similar to HDAC inhibitors as was sorafenib. Therefore, the CMap analysis indicated that inhibition of HDAC may be a novel and specific function of sorafenib.

### Sorafenib indirectly inhibits HDAC activity in both sorafenib-sensitive and -resistant HCC cells

**Figure 3:**
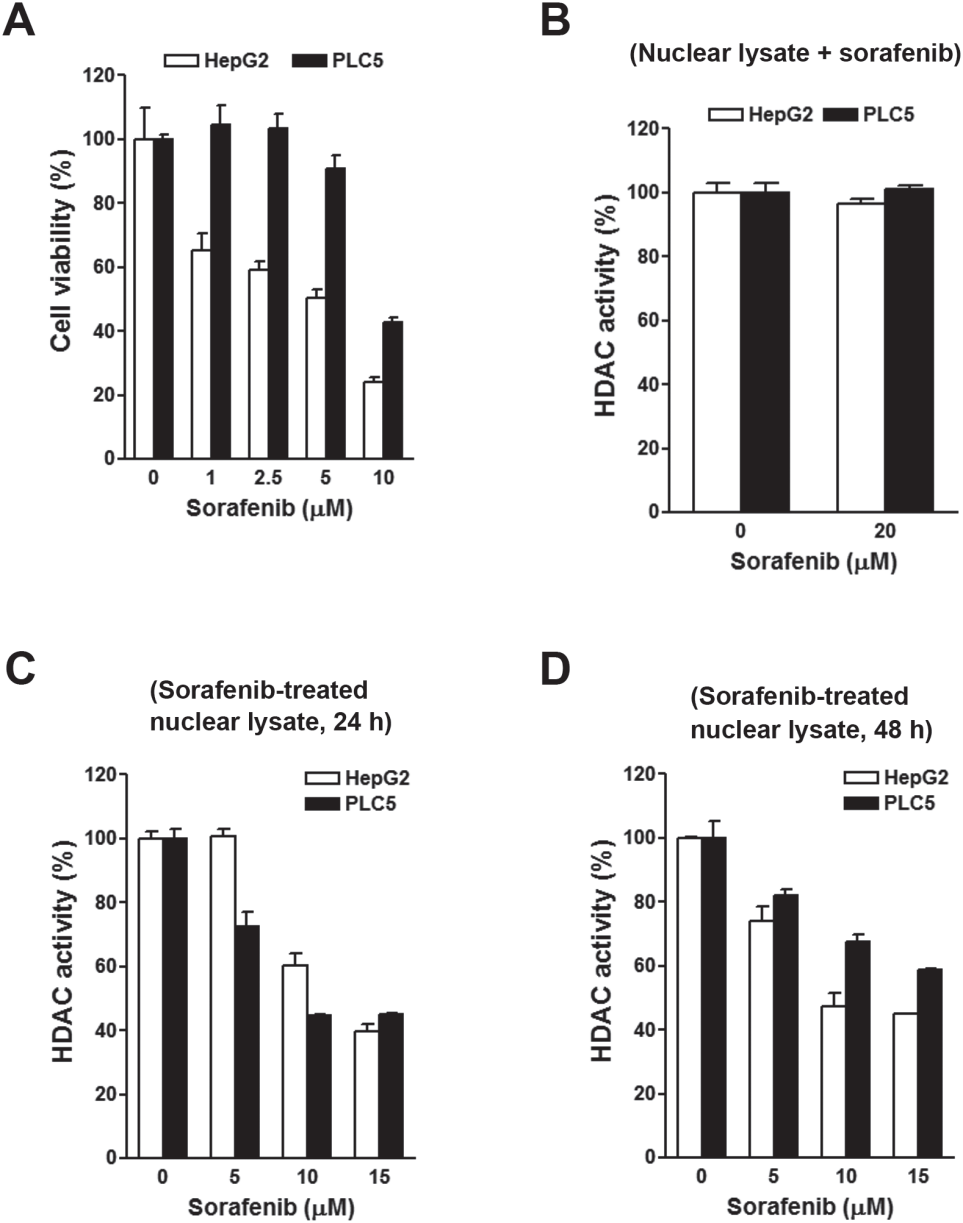
Effects of sorafenib on the cell viability and histone deacetylase (HDAC) activity in HepG2 and PLC5 cells **(A)** HepG2 and PLC5 cells were treated with 0∼10 μM sorafenib for 72 h. Cell viability was examined by an MTT assay. **(B)** Nuclear lysates of HepG2 and PLC5 cells were incubated with 20 μM sorafenib for 0.5 h. Then an *in vitro* HDAC activity assay was performed. **(C, D)** HepG2 and PLC5 cells were treated with 0∼10 μM sorafenib for 24 h **(C)** and 48 h **(D)**. Nuclear lysates were prepared for an *in vitro* HDAC activity assay.

**Figure 4:**
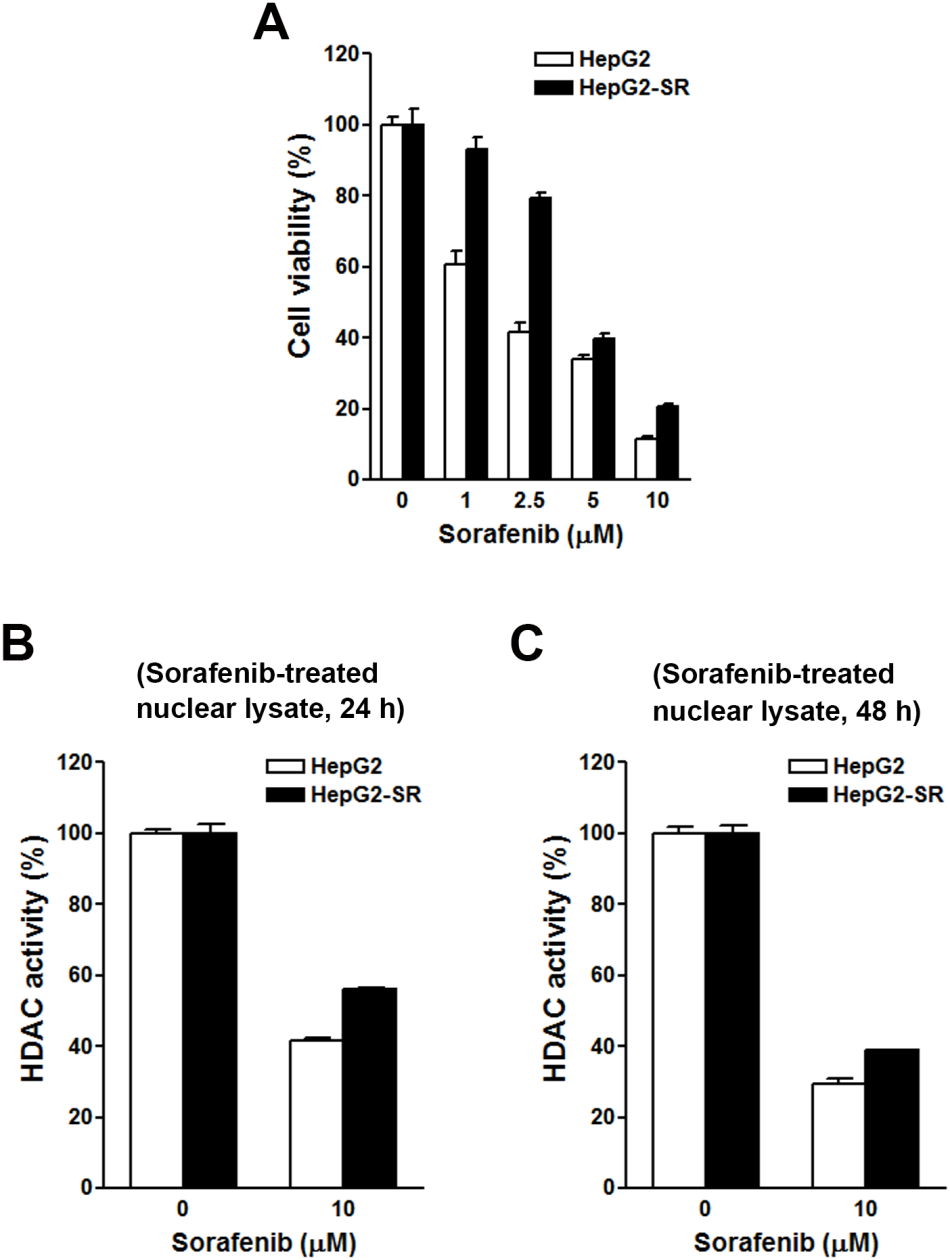
Effects of sorafenib on cell viability and histone deacetylase (HDAC) activities in HepG2 and HepG2-SR cells **(A)** HepG2 and HepG2-SR cells were treated with 0∼10 μM sorafenib for 72 h. Cell viability was examined by an MTT assay. **(B, C)** HepG2 and HepG2-SR cells were treated with 10 μM sorafenib for 24 h **(B)** and 48 h **(C)**. Nuclear lysates were prepared for an *in vitro* HDAC activity assay.

To investigate the effects of sorafenib on HDAC activity, two HCC cell lines, HepG2 and PLC/PRF/5 (PLC5), were used. First, their sensitivities to sorafenib were determined by an MTT cell viability assay. As shown in Figure 3A, HepG2 cells were more sensitive to sorafenib than PLC5 cells, suggesting primary resistance of PLC5 cells to sorafenib. To test whether sorafenib can directly inhibit HDAC activity, nuclear lysates of HepG2 and PLC5 cells were incubated with sorafenib *in vitro*, and then an HDAC activity assay was performed. However, sorafenib did not inhibit HDAC activity (Figure 3B). Next, we examined if sorafenib could indirectly inhibit HDAC activity. HepG2 and PLC5 cells were treated with sorafenib for 24 and 48 h, and then nuclear lysates were prepared for an HDAC activity assay. As shown in Figure 3C, sorafenib significantly inhibited HDAC activity at 24 and 48 h. We noted that the inhibitory effect of sorafenib on HDAC activity in PLC5 cells was slightly attenuated at 48 h (Figure 3D), which implied that the HDAC-inhibitory ability of sorafenib was partially affected by the primary sorafenib-resistant property of cancer cells. To confirm this phenomenon, a HepG2 (HepG2-SR) cell line with sorafenib-acquired resistance was generated. A cell viability analysis demonstrated the resistance of HepG2-SR cells to sorafenib (Figure 4A). Sorafenib was able to inhibit HDAC activity in both HepG2 and HepG2-SR cells at 24 and 48 h (Figure 4B, 4C). Similarly, slight resistance of HepG2-SR cells to sorafenib-induced HDAC inhibition at 24 and 48 h was also observed (Figure 4B, 4C). Therefore, sorafenib inhibits HDAC activity in both sorafenib-sensitive and -resistant HCC cells, although this effect was partially affected by primary and acquired drug resistance of cancer cells.

### Clinical intervention with the HDAC-inhibitory activity of sorafenib in HCC

**Figure 5:**
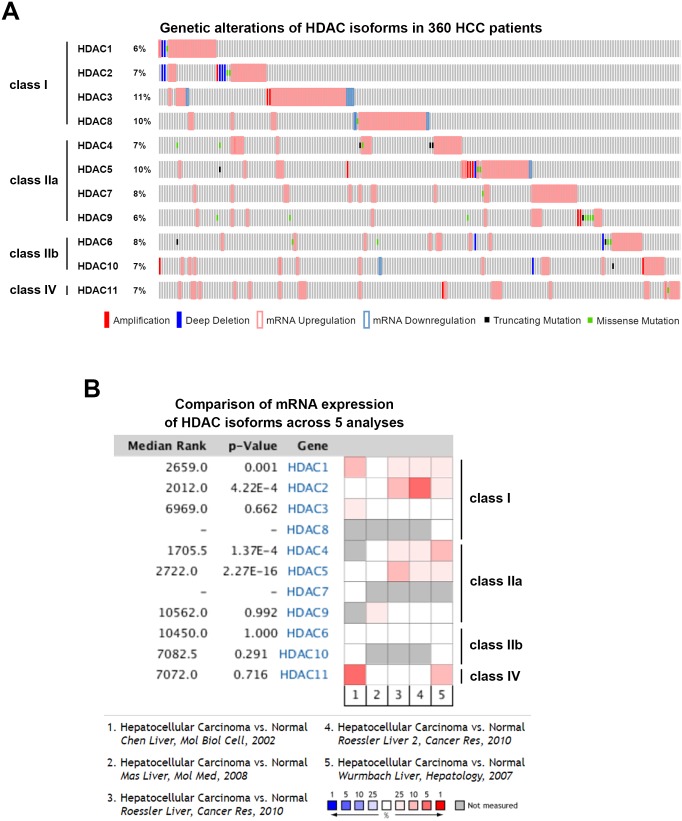
Cancer genomics and Oncomine analysis of genetic alterations of histone deacetylases (HDACs) **(A)** Genetic alterations of class I, IIa/b, and IV HDACs were analyzed using the online tool, cBioPortal. **(B)** Oncomine analysis of mRNA expressions of HDAC isoforms in hepatocellular carcinoma (HCC) was performed as described in “Materials and Methods”.

**Figure 6:**
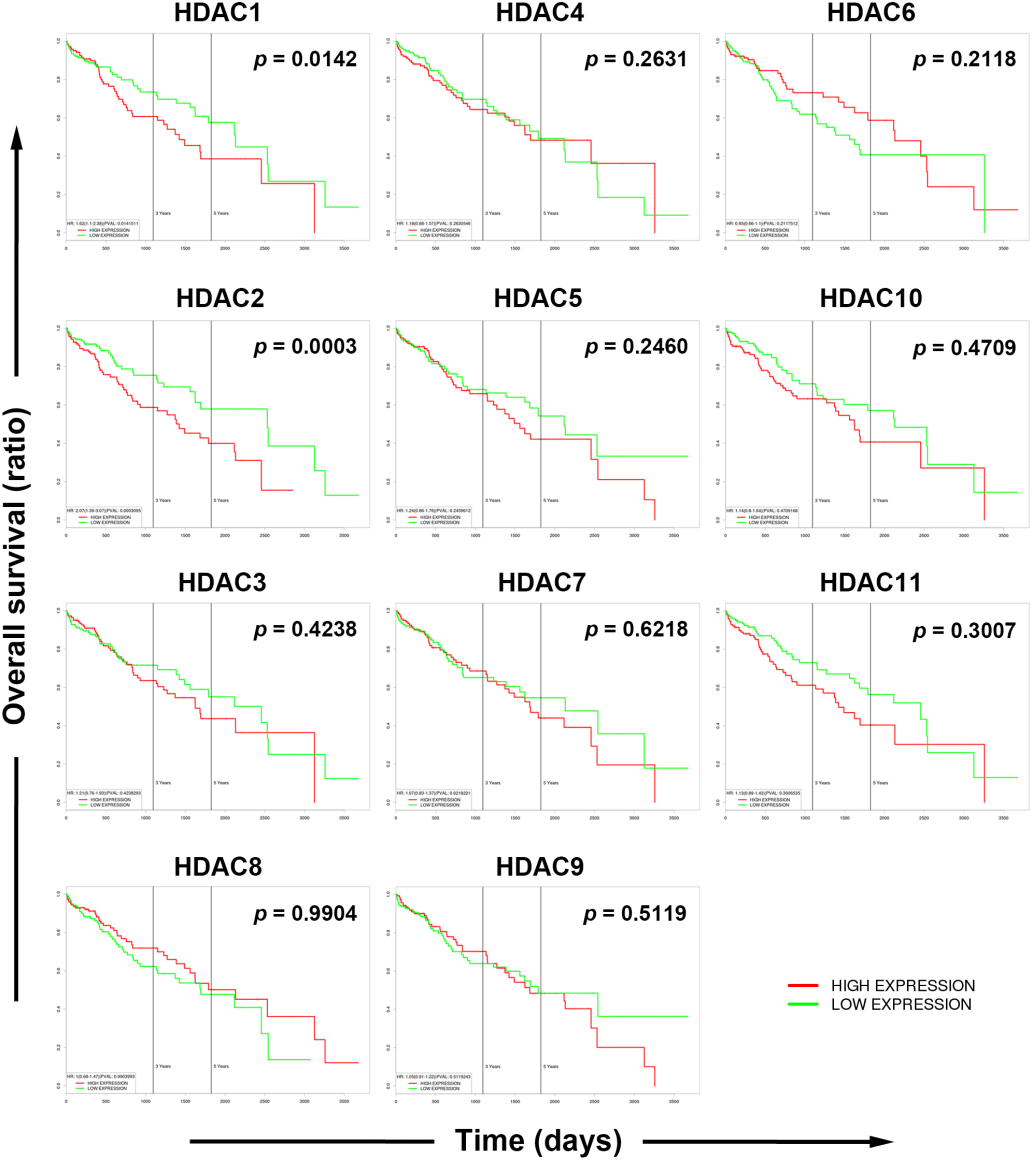
Role of each histone deacetylase (HDAC) isoform in the overall survival of hepatocellular carcinoma (HCC) patients Overall survival of HCC patients with high or low expression of each HDAC isoform was analyzed using the online tool, PROGgeneV2.

To gain more insights into the clinical benefits of sorafenib through its HDAC inhibition, genetic alterations, including the mutation status, copy number alterations, and mRNA expressions, were examined by accessing the cBioPortal for Cancer Genomics (http://www.cbioportal.org/) [10, 11]. There are 18 HDACs identified in humans, which are classified based on homologies to yeast HDACs [12, 13]. Classes I (HDACs 1, 2, 3, and 8), IIa (HDACs 4, 5, 7, and 9), IIb (HDACs 6 and 10), and IV (HDAC11) are zinc-dependent deacetylases [12, 13]. Class III HDACs (sirtuins 1∼7) are zinc-independent and require NAD^+^ for their activities [14]. Herein, we focused on zinc-dependent deacetylases. As shown in Figure 5A, 58% of HCC patients showed genetic alterations of class I, IIa/b, and IV HDACs. The most common alteration of HDACs was mRNA upregulation. To confirm this result, another five HCC patient datasets [15–18] were used to compare mRNA overexpressions of HDAC isoforms by mining the Oncomine database (http://www.oncomine.org/) [19]. As shown in Figure 5B, mRNAs of HDAC1, HDAC2, HDAC4, and HDAC5 were significantly upregulated in these datasets. To further investigate the essential role of each HDAC isoform in the overall survival of HCC patients, a Kaplan-Meier (KM) survival analysis using the PROGgeneV2 prognostic database (http://www.compbio.iupui.edu/proggene/) [20] was performed. Interestingly, only patients with high expression of HDAC1 or HDAC2 had shorter overall survival (Figure 6). These results indicated that inhibition of HDAC1 and HDAC2 may provide clinical benefits for HCC patients.

### Connectivity analysis between sorafenib and HDAC isoforms

**Figure 7:**
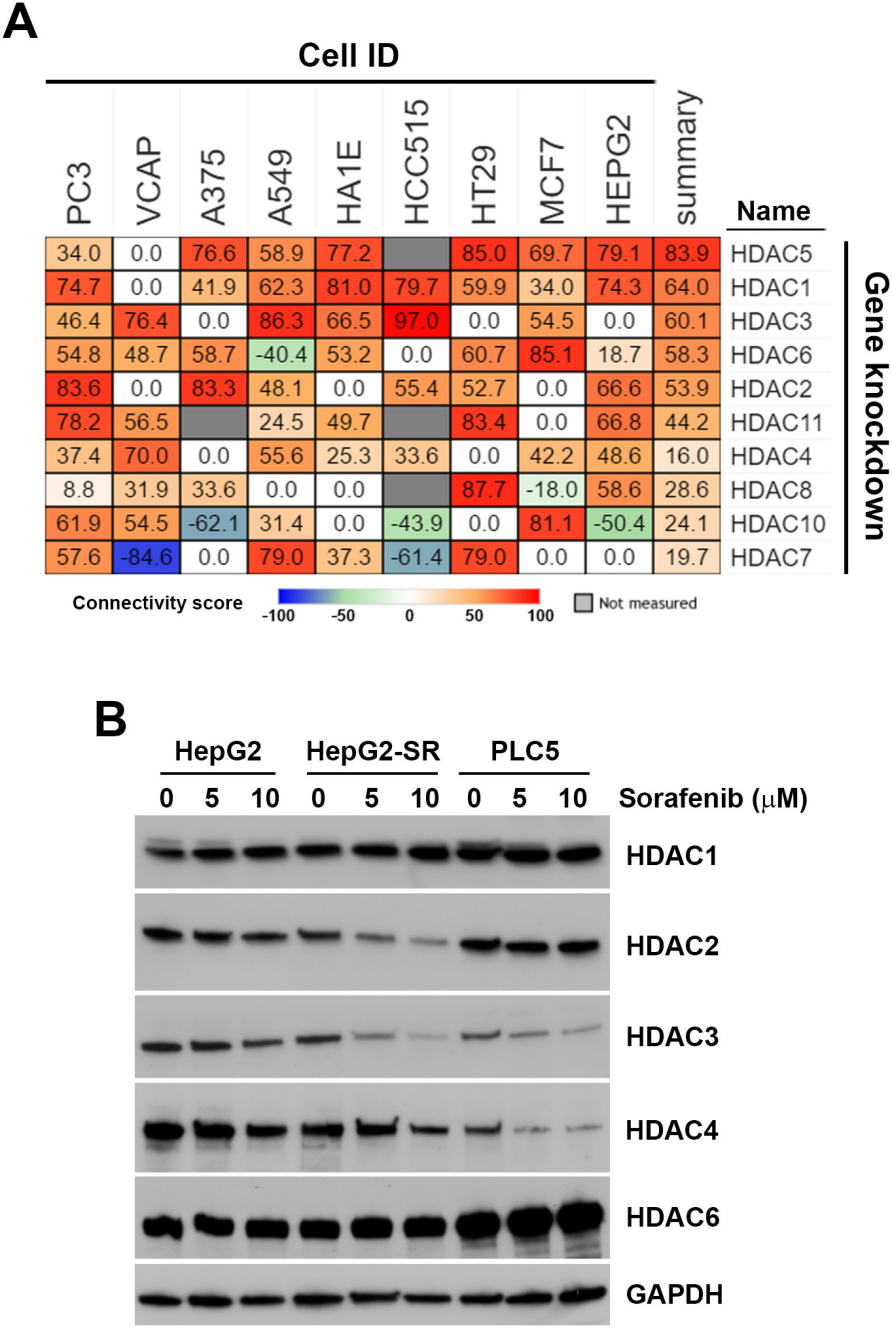
Connectivity analysis of sorafenib with each histone deacetylase (HDAC) isoform **(A)** Connections of sorafenib with the knockdown of each HDAC isoform (except HDAC9) were analyzed using an online Touchstone tool of the CLUE database as described in “Materials and Methods”. Sorafenib-HDAC isoform connections were viewed as a heatmap ranked by the summary connectivity score. **(B)** HepG2, HepG2-SR, and PLC5 cells were treated with 0∼10 μM sorafenib for 24 h. Protein expressions of HDAC1, HDAC2, HDAC3, HDAC4, and HDAC6 were analyzed by Western blotting.

To investigate the drug-gene connectivity of sorafenib and HDAC isoforms, gene expression profiles of sorafenib-treated cells were compared to those in specific HDAC isoform-knockdown cells. As shown in Figure 7A, sorafenib-HDAC connectivity varied among these cells. In general, knockdown of HDAC1, HDAC3, and HDAC5 had similar gene expression profiles (summary connectivity score > 60) to sorafenib. In HepG2 cells, however, knockdown of HDAC1, HDAC2, HDAC5, and HDAC11 had higher connectivity scores (> 50). These results indicated that sorafenib may have the ability to inhibit HDAC1 and HDAC2 in HCC cells.

To investigate whether sorafenib can downregulate HDAC expression, protein levels of HDAC1, HDAC2, HDAC3, HDAC4, and HDAC6 in sorafenib-treated HepG2, HepG2-SR, and PLC5 cells were analyzed by Western blotting. As shown in Figure 7B, expressions of HDAC1 and HDAC6 were not altered by sorafenib in these cells. Expressions of HDAC2, HDAC3, and HDAC4 were reduced by sorafenib in both HepG2 and HepG2-SR cells. PLC5 cells had lower expressions of HDAC3 and HDAC4, which were further reduced by sorafenib. Therefore, sorafenib selectively downregulated protein expressions of HDAC isoforms.

## DISCUSSION

It is currently accepted that polypharmacology (one drug interacts with multiple targets) is a general property of small molecules. Polypharmacological studies can be applied not only for drug repurposing (new uses for old drugs) but also for identifying novel mechanisms of drugs [6]. Gene expression profiling for drug connectivity in large-scale perturbation databases, such as CMap and LINCS, provides enormous opportunities for exploring polypharmacology [21]. For example, we previously performed a LINCS analysis to demonstrate functional disparities between two structurally and mechanistically similar DNA-hypomethylating agents, azacytidine and decitabine [22]. In addition, we identified novel action mechanisms of a Chinese herbal medicine, berberine, and repurposed the cyclin-dependent kinase inhibitor, GW8510, as a ribonucleotide reductase M2 inhibitor by CMap analyses [23, 24].

Sorafenib is a multi-kinase inhibitor that inhibits cell surface tyrosine kinase receptors (such as VEGFR and platelet-derived growth factor receptor (PDGFR)) and downstream intracellular serine/threonine kinases (such as RAF family kinases) [25]. Our analysis using CLUE indeed predicted kinase targets of sorafenib. Interestingly, HDACs were also predicted to be molecular targets of sorafenib. An *in vitro* experimental analysis showed that sorafenib indirectly inhibited HDAC activities through downregulating protein expressions. Overexpressions of HDACs were found in tumors, leading to the epigenetic silencing of tumor-suppressor genes. Therefore, inhibition of HDACs is considered a potential strategy for treating cancers through reactivating tumor-suppressor genes [26]. Our analysis using the cBioPortal for Cancer Genomics also indicated the frequent upregulation of HDAC mRNAs in HCC. Specifically, HDAC1 and HDAC2 upregulation was associated with poor overall survival of HCC patients. Therefore, the HDAC-inhibitory property of sorafenib may cause epigenetic changes in gene expressions and provide clinical benefits for HCC patients through reactivating tumor-suppressor genes.

The mechanism for inhibiting HDAC activities by sorafenib was not resolved in the current study. Direct *in vitro* incubation of sorafenib with nuclear cell lysates did not reduce HDAC activities, suggesting that sorafenib indirectly inhibits HDAC activities. The substrate used for the *in vitro* HDAC activity assay was Ac-Lys(Ac)-pNA. Theoretically, this assay can detect all HDACs that can deacetylate histone. Sorafenib selectively downregulated HDAC protein expressions; however, this seemed to incompletely explain the actions of sorafenib, because protein expressions of HDACs examined in this study were only partially reduced by sorafenib in HepG2 cells. Therefore, other mechanisms may exist. Phosphorylation of HDACs is important for their activation [27]. For example, HDAC1 and HDAC2 are phosphorylated by casein kinase 2 (CK2) [28–30]. Because sorafenib is a multi-kinase inhibitor, we hypothesized that it may inhibit HDAC kinase activities and then inhibit HDAC activities.

Although sorafenib can extend the median survival time of HCC patients by about 3 months [3, 4], drug resistance usually develops. A better understanding of the underlying mechanisms will help develop therapeutic strategies for overcoming sorafenib resistance. Our study indicated that inhibition of HDAC activities is associated with the anticancer activity of sorafenib, and loss of this effect partially contributes to the development of sorafenib resistance. In other words, amplification of the HDAC-inhibitory activities of sorafenib might be able to enhance its anticancer activity and overcome drug resistance. Indeed, previous studies demonstrated that HDAC inhibitors can enhance the anti-HCC activity of sorafenib [31, 32]. Furthermore, novel combination treatments with herbal medicines or approved drugs which can inhibit HDAC activity could also be a promising strategy, because these agents are less-toxic and more-safe. For example, *Ginkgo biloba* extracts, retrieved from the Chinese herbal medicine, displayed HDAC-inhibitory activity [33]. A recent clinical trial indicated that a combination of *Ginkgo biloba* extracts with sorafenib was safe and tolerable for advanced HCC patients and slightly improved their overall survival [34]. Statins, the cholesterol-lowering drugs prescribed to prevent heart attacks, inhibits HDAC activity and overcomes the hypoxic resistance of HCC cells to sorafenib [35, 36]. Our previous study also showed that methotrexate, an anti-folate drug for treating cancers and autoimmune diseases, can inhibit HDAC activity [37]. Synergistic anticancer effect of methotrexate and sorafenib in HCC has also been reported recently [38]. Therefore, based on our observations that HDACs are novel targets for sorafenib, drug combinations focusing on HDACs may be an effective strategy for managing HCC. However, it should be noted that the direct relationship between HDAC inhibition and the synergistic anticancer activity of sorafenib and HDAC-targeting drugs has not been clearly demonstrated in these studies.

In summary, our study demonstrated that gene expression profiling using large-scale drug connectivity databases, such as CMap and LINCS, is highly useful for polypharmacological studies. By comparing drug connectivity profiles with sorafenib, we identified that inhibition of HDAC activities was associated with the anticancer activities of sorafenib in HCC cells. Our study provides a novel aspect of sorafenib for treating HCC and a novel strategy to study polypharmacology. However, there were still limitations in our study. For example, the mechanism(s) and the clinical benefits of the HDAC-inhibitory effect of sorafenib were not fully elucidated. These issues should be addressed in future investigations.

## MATERIALS AND METHODS

### CMap analysis

Connections of sorafenib and protein kinase inhibitors to other compounds or sorafenib to HDAC isoform knockdown were directly obtained from the CLUE database (https://clue.io/) using the “Touchstone” online tool (May 4, 2017, date last accessed). The “Perturbational Class” was set to “CMap Class” for searching drugs similar to sorafenib and protein kinase inhibitors. Drug connections (type: PCL) can be viewed as a heatmap. The option “Strong connections” was selected. For the connectivity analysis of sorafenib and HDAC isoform knockdown, the perturbational type was set to “Gene Knock-Down”, and a name filter was set to visualize the heatmap of HDAC isoforms.

### The Cancer Genome Atlas (TCGA) analysis

A cancer genomics analysis was performed (May 4, 2017, date last accessed) by querying the online cBioPortal for Cancer Genomics (http://www.cbioportal.org/) [10, 11]. The dataset “Liver Hepatocellular Carcinoma (TCGA, Provisional)” was used. Genomic profiles including mutations, putative copy-number alterations, and mRNA expressions (with z-scores = ±2) were selected for querying HDACs. Results are shown as OncoPrint. The overall survival of HCC patients (TCGA dataset) with high or low HDAC isoform expression was analyzed using the PROGgeneV2 database (http://www.compbio.iupui.edu/proggene/) [20].

### Oncomine analysis

Oncomine (http://www.oncomine.org/) is a collection of cancer microarray databases with a web-based data-mining platform [19]. To investigate the mRNA expression of each HDAC isoform, a comparison of the transcriptome data in hepatocellular carcinoma with respect to normal tissues was performed. Thresholds for significance were multiple of expression > 2, *p* value < 0.05, and ranking of gene in the analyses > top 10%. Red signifies gene overexpression, and blue signifies gene underexpression. The intensity of the color signifies the best ranking of genes in those analyses.

### Materials

Fetal bovine serum (FBS) was purchased from Gibco. Dulbecco's modified Eagle medium (DMEM), L-glutamine, sodium pyruvate, non-essential amino acids (NEAAs), and an antibiotic-antimycotic (penicillin G, streptomycin, and amphotericin B) were purchased from Life Technologies. HDAC1, HDAC2, HDAC3, and HDAC4 antibodies were purchased from Cell Signaling. The GAPDH antibody was purchased form GeneTex. Horseradish peroxidase-labeled goat anti-rabbit and anti-mouse secondary antibodies were purchased from Jackson ImmunoResearch. Sorafenib was purchased from Cayman Chemical. Dimethyl sulfoxide (DMSO) and 3-(4,5-dimethylthiazol-2-yl)-2,5-diphenyl tetrazolium bromide (MTT) were purchased from Sigma. Protease and phosphatase inhibitor cocktails were purchased from Roche. Other chemicals or reagents were purchased from OneStar Biotechnology.

### Cell culture

HepG2 and PLC/PRF/5 (PLC5) cells were purchased from the Bioresources Collection and Research Center (BCRC), Food Industry Research and Development Institute (Hsinchu, Taiwan). Sorafenib-resistant HepG2-SR cells were established by repeatedly exposing them to an increasing dose of sorafenib for several months. Cells were cultured in DMEM supplemented with 10% FBS, 1 mM sodium pyruvate, 1% L-glutamine, 1% NEAAs, and a 1% antibiotic-antimycotic solution, and incubated at 37 °C in a humidified incubator containing 5% CO_2_.

### Cell viability assay

Cell viability was examined with an MTT assay. Briefly, cells were spread on 96-well plates and treated with drugs for 72 h. Then, 0.5 mg/mL of MTT was added to each well, and cells were cultured for an additional 4 h. The blue MTT formazan precipitates were dissolved in 200 μL of DMSO. The absorbance at 570 nm was measured using a multi-well plate reader.

### HDAC activity assay

Nuclear lysates with or without sorafenib treatment were prepared using a Nuclear/Cytosol Fractionation Kit (BioVision) according to the manufacturer's instructions. Pan-HDAC activity was measured with an HDAC Activity Colorimetric Assay Kit (BioVision). Nuclear lysates were incubated at 37°C with or without sorafenib, and the HDAC reaction was initiated by adding the Ac-Lys(Ac)-pNA substrate. After 3 h, lysine developer was added, and the mixture was incubated for another 30 min. The absorbance at 405 nm was measured using a multi-well plate reader.

## SUPPLEMENTARY MATERIALS FIGURE AND TABLE



## Abbreviations

CMap: Connectivity Map
HCC: hepatocellular carcinoma
HDAC: histone deacetylase
HepG2-SR: sorafenib-resistant HepG2 cells
LINCS: The Library of Integrated Cellular Signatures
PDGFR: platelet-derived growth factor receptor
PLC5: PLC/PRF/5
VEGFR: vascular endothelial growth factor receptor
