# Supplementary material for: In silico and in vitro identification of inhibitory activities of sorafenib on histone deacetylases in hepatocellular carcinoma cells
Source: Oncotarget. 2017 Sep 16;8(49):86168–80. doi: 10.18632/oncotarget.21030 (PMC5689675; doi:10.18632/oncotarget.21030)
Supplement: Supplementary file 1 [file oncotarget-08-86168-s001.pdf]

In silico and *in vitro* identification of inhibitory activities of sorafenib on histone deacetylases in hepatocellular carcinoma cells

SUPPLEMENTARY MATERIALS

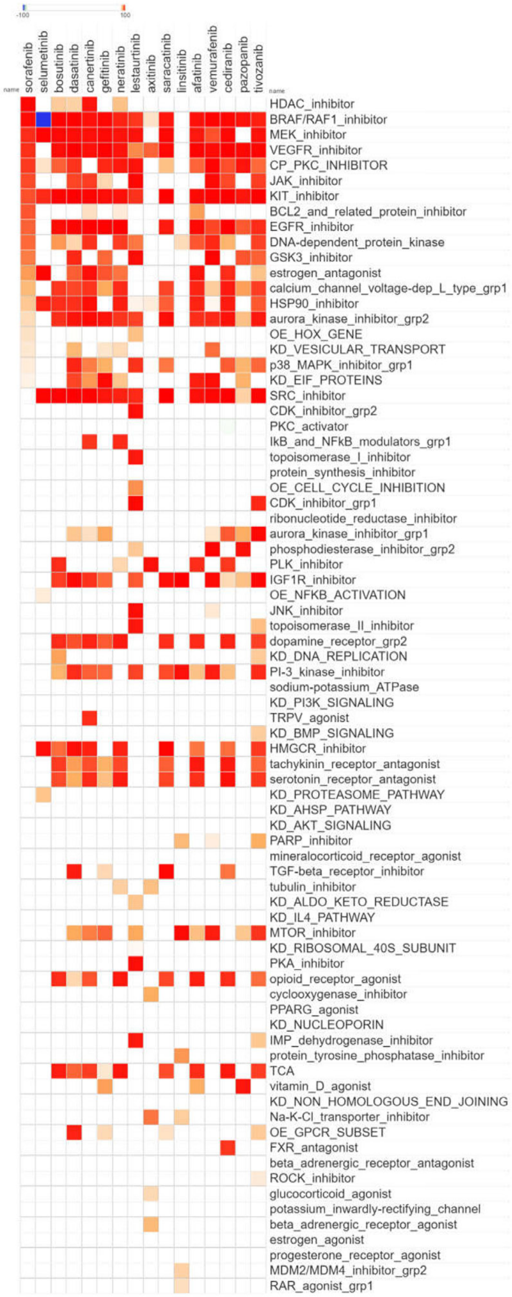

Supplementary Figure 1: Original image file for Figure 2.

Supplementary Table 1: A list of protein kinase inhibitors used for CMap analysis in Figure 2

Drug name	Description
Afatinib	EGFR inhibitor
Axitinib	PDGFR inhibitor
Bosutinib	ABL inhibitor
Canertinib	EGFR inhibitor
Cediranib	KIT inhibitor
Dasatinib	BCR-ABL inhibitor
Gefitinib	EGFR inhibitor
Lestaurtinib	FLT3 inhibitor
Linsitinib	IGF-1 inhibitor
Neratinib	EGFR inhibitor
Pazopanib	KIT inhibitor
Saracatinib	SRC inhibitor
Selumetinib	MEK inhibitor
Tivozanib	VEGFR inhibitor
Vemurafenib	RAF inhibitor
